# Supplementary material for: Can multitrophic interactions shape morphometry, allometry, and fluctuating asymmetry of seed-feeding insects?
Source: PLoS One. 2020 Nov 11;15(11):e0241913. doi: 10.1371/journal.pone.0241913 (PMC7657534; doi:10.1371/journal.pone.0241913)
Supplement: S1 Table — (DOCX) [file pone.0241913.s001.docx]

**S1 Table. Model testing the existence or not of fluctuating asymmetry with different combinations of random effects for the intercept and slope, by testing mixed models with restricted likelihood (REML).**

| **M1= measure ~ side * (SB + FI +PR) + (1\|ind)** |
| --- |
| **M2= measure ~ side * (SB+ FI +PR) + (side\|ind)** |
| **M3= measure ~ side * (SB + FI +PR) + (side:FI\|ind)** |
| **M4= measure ~ side * (SB + FI +PR) + (side:PR\|ind)** |

ind = measurement error, M1= without asymmetry, M2= Different slopes to each side, M3= Different slope to each side in each infestation categories, M4= Different slope to each side in each parasitism categories. SB=seed biomass, FI= fruit infestation, PR= parasitism rate.
