# Supplementary material for: Can multitrophic interactions shape morphometry, allometry, and fluctuating asymmetry of seed-feeding insects?
Source: PLoS One. 2020 Nov 11;15(11):e0241913. doi: 10.1371/journal.pone.0241913 (PMC7657534; doi:10.1371/journal.pone.0241913)
Supplement: S2 Table — (DOCX) [file pone.0241913.s002.docx]

S2 Table. Allometric coefficient with slope value, confidence interval according to the categories and morphological structures of *Merobruchus terani*.

| Categories | Trait | Slope | CI 2.5 | CI 97.5 | Nº of individuals | Subcategories |
| --- | --- | --- | --- | --- | --- | --- |
| Seed Infestation |  |  |  |  |  |  |
|  |  | 0.153 | 0.124 | 0.182 | 323 | Low |
|  | Elytra | 0.127 | 0.065 | 0.190 | 191 | Medium |
|  |  | - | - | - | - | High |
|  |  | 0.080 | 0.043 | 0.118 | 323 | Low |
|  | Pronotum | 0.168 | 0.076 | 0.262 | 191 | Medium |
|  |  |  | - | - | - | High |
|  | | | | | |  |
| Parasitism rate |  | 0.161 | 0.132 | 0.190 | 432 | Low |
|  | Elytra | 0.153 | 0.093 | 0.215 | 71 | Medium |
|  |  | 0.061 | -0.025 | 0.148 | 15 | High |
|  |  | 0.111 | 0.073 | 0.150 | 432 | Low |
|  | Pronotum | 0.033 | -0.054 | 0.123 | 71 | Medium |
|  |  | -0.033 | -0.158 | 0.090 | 15 | High |
|  | | | | | |  |
| Seed Biomass |  | 0.138 | 0.096 | 0.181 | 183 | Small |
|  | Elytra | 0.126 | 0.077 | 0.175 | 181 | Medium |
|  |  | 0.111 | 0.059 | 0.163 | 154 | Large |
|  |  | 0.093 | 0.043 | 0.145 | 183 | Small |
|  | Pronotum | 0.092 | 0.030 | 0.155 | 181 | Medium |
|  |  | 0.0921 | -0.015 | 0.201 | 154 | Large |
