# Supplementary material for: Can multitrophic interactions shape morphometry, allometry, and fluctuating asymmetry of seed-feeding insects?
Source: PLoS One. 2020 Nov 11;15(11):e0241913. doi: 10.1371/journal.pone.0241913 (PMC7657534; doi:10.1371/journal.pone.0241913)
Supplement: S3 Table — (DOCX) [file pone.0241913.s003.docx]

S3 Table. Allometric coefficient with slope value, confidence interval according to the categories and morphological structures of *Stator maculatopygus*.

| Categories | Trait | | | Slope | | CI 2.5 | | CI 97.5 | Nº of individuals | | | | Subcategories |
| --- | --- | --- | --- | --- | --- | --- | --- | --- | --- | --- | --- | --- | --- |
| Seed Infestation |  | | |  | |  | |  |  | | |  | |
|  |  | | | 0.058 | | 0.013 | | 0.103 | 45 | | | Low | |
|  | Elytra | | | *-* | | *-* | | *-* | 4 | | | Medium | |
|  |  | | | *-* | *-* | | | *-* | 0 | | | High | |
|  |  | | | 0.085 | | 0.011 | | 0.160 | | 45 | Low | | |
|  | Pronotum | | | **-** | **-** | | | **-** | | 4 | Medium | | |
|  |  | | **-** | | **-** | | | **-** | | 0 | High | | |
|  | | | | | | | | | | |  | | |
| Parasitism rate |  | | | 0.050 | | 0.014 | 0.087 | | | 43 | Low | | |
|  | Elytra | | | *-* | | *-* | *-* | | | 4 | Medium | | |
|  |  | | | *-* | | *-* | *-* | | | 3 | High | | |
|  |  | | | 0.033 | | -0.025 | 0.092 | | | 43 | Low | | |
|  | Pronotum | | | **-** | | **-** | **-** | | | 4 | Medium | | |
|  |  | | - | | | - | - | | | 3 | High | | |
|  | | | | | | | | | | |  | | |
| Seed Biomass | |  | | 0.037 | | -0.033 | | 0.108 | | 8 | Small | | |
|  |  | Elytra | | 0.105 | | 0.013 | | 0.199 | | 11 | Medium | | |
|  |  |  | | 0.029 | | -0.026 | | 0.084 | | 31 | Large | | |
|  |  |  | | 0.025 | | -0.077 | | 0.127 | | 8 | Small | | |
|  |  | Pronotum | | 0.100 | | -0.052 | | 0.257 | | 11 | Medium | | |
|  |  |  | | 0.086 | | -0.014 | | 0.187 | | 31 | Large | | |
