# Supplementary material for: Can multitrophic interactions shape morphometry, allometry, and fluctuating asymmetry of seed-feeding insects?
Source: PLoS One. 2020 Nov 11;15(11):e0241913. doi: 10.1371/journal.pone.0241913 (PMC7657534; doi:10.1371/journal.pone.0241913)
Supplement: S4 Table — (DOCX) [file pone.0241913.s004.docx]

S4 Table. Allometric coefficient with slope value, confidence interval according to the categories and morphological structures of *Allorhogasvulgaris*.

| Categories | Trait | Slope | CI 2.5 | CI 97.5 | | | Nº of individuals | | Subcategories | |  |
| --- | --- | --- | --- | --- | --- | --- | --- | --- | --- | --- | --- |
| Seed Infestation |  |  |  |  | |  | |  | |  | |
|  |  | 0.435 | 0.222 | 0.689 | | 34 | | Low | |  | |
|  | Wing | 0.556 | 0.412 | 0.719 | | 51 | | Medium | |  | |
|  |  | 0.712 | 0.537 | 0.921 | | 61 | | High | |  | |
|  |  | 0.458 | 0.235 | 0.726 | | 34 | | Low | |  | |
|  | Tibia | 0.496 | 0.321 | 0.700 | | 51 | | Medium | |  | |
|  |  | 0.769 | 0.511 | 1.112 | | 61 | | High | |  | |
|  | | | | | | | |  | |  | |
| Parasitism rate |  | 0.608 | 0.499 | | 0.728 | 114 | | Low | |  | |
|  | Wing | 0.464 | 0.198 | | 0.798 | 28 | | Medium | |  | |
|  |  | - | - | | - | 0 | | High | |  | |
|  |  | 0.575 | 0.441 | | 0.727 | 114 | | Low | |  | |
|  | Tibia | 0.497 | 0.129 | | 1.018 | 28 | | Medium | |  | |
|  |  | - | - | | - | 0 | | High | |  | |
|  | | | | | | | |  | |  | |
| Seed Biomass |  | 0.600 | 0.439 | 0.789 | | 61 | | Small | |  | |
|  | Wing | 0.502 | 0.358 | 0.664 | | 62 | | Medium | |  | |
|  |  | 0.702 | 0.472 | 0.995 | | 23 | | Large | |  | |
|  |  | 0.647 | 0.479 | 0.846 | | 61 | | Small | |  | |
|  | Tibia | 0.464 | 0.259 | 0.708 | | 62 | | Medium | |  | |
|  |  | 0.617 | 0.259 | 1.145 | | 23 | | Large | |  | |
