# Supplementary material for: Can multitrophic interactions shape morphometry, allometry, and fluctuating asymmetry of seed-feeding insects?
Source: PLoS One. 2020 Nov 11;15(11):e0241913. doi: 10.1371/journal.pone.0241913 (PMC7657534; doi:10.1371/journal.pone.0241913)
Supplement: S5 Table — All models have the same structure with different random factors. In which, M1 is the model with no difference in sides; M2 with different slopes for each side; M3 with different slopes for each side in infestation categories; M4 different slopes for each side in parasitism categories. (DOCX) [file pone.0241913.s005.docx]

S5 Table. Result comparing the fluctuating asymmetry models of *Merobruchus terani* and *Stator. maculatopygus* elytra and *Allorhogas vulgaris’* wing and tibia. All models have the same structure with different random factors. In which, M1 is the model with no difference in sides; M2 with different slopes for each side; M3 with different slopes for each side in infestation categories; M4 different slopes for each side in parasitism categories.

| Species | Trait | | | Model | d.f | Loglink | d.f | Chisq | P |
| --- | --- | --- | --- | --- | --- | --- | --- | --- | --- |
|  | |  | | M1 | 14 | 1634.3 |  |  |  |
| *M. terani* | | Elytra | | M2 | 16 | 1634.3 | 2 | 0 | 1 |
|  | |  | | M3 | 19 | 1634.3 | 3 | 0 | 1 |
|  | |  | | M4 | 41 | 1634.3 | 22 | 0 | 1 |
|  | | |  | M1 | 14 | 320.16 |  |  |  |
| *S. maculatopygus* | | | Elytra | M2 | 16 | 320.16 | 2 | 0 | 1 |
|  | | |  | M3 | 19 | 320.16 | 3 | 0 | 1 |
|  | | |  | M4 | 41 | 320.16 | 22 | 0 | 1 |
|  | | |  |  |  |  |  |  |  |
|  | | |  | M1 | 14 | 504.99 |  |  |  |
|  | | | Wing | M2 | 16 | 504.99 | 2 | 0 | 1 |
|  | | |  | M3 | 19 | 504.99 | 3 | 0 | 1 |
| 1. *vulgaris* | | |  | M4 | 41 | 504.99 | 22 | 0 | 1 |
|  | | |  | M1 | 14 | 1489.8 |  |  |  |
|  | | | Tibia | M2 | 16 | 1489.8 | 2 | 0 | 1 |
|  | | |  | M3 | 19 | 1489.8 | 3 | 0 | 1 |
|  | | |  | M4 | 41 | 1489.8 | 22 | 0 | 1 |
