# Supplementary material for: Can multitrophic interactions shape morphometry, allometry, and fluctuating asymmetry of seed-feeding insects?
Source: PLoS One. 2020 Nov 11;15(11):e0241913. doi: 10.1371/journal.pone.0241913 (PMC7657534; doi:10.1371/journal.pone.0241913)
Supplement: S6 Table — Results are displayed in comparison to the right elytra. (DOCX) [file pone.0241913.s006.docx]

S6 Table. Fluctuating asymmetry between left and right sides for elytra length of *Merobruchus terani,* according to categories of seed biomass, fruit infestation and parasitism rate. Results are displayed in comparison to the right elytra.

| *M. terani* X Elytra | Estimate | SD | d.f. | T | P |
| --- | --- | --- | --- | --- | --- |
| **(Intercept)** | 1.44 | 0.01 | 3096 | 121.29 | **<0.001*** |
| Left side | 0.01 | 0.02 | 3096 | 0.35 | 0.73 |
| Medium seed | -0.08 | 0.01 | 3096 | -9.12 | **<0.001*** |
| Small seed | -0.12 | 0.01 | 3096 | -13.28 | **<0.001*** |
| Infestation rate | 0.2 | 0.03 | 3096 | 6.09 | **<0.001*** |
| Medium parasitism rate | 0.01 | 0.01 | 3096 | 1.35 | 0.18 |
| High parasitism rate | 0.02 | 0.02 | 3096 | 0.98 | 0.33 |
| Left side: medium seed | 0 | 0.01 | 3096 | -0.07 | 0.94 |
| Left side: small seeds | 0 | 0.01 | 3096 | -0.06 | 0.95 |
| Left side: infestation rate | 0 | 0.05 | 3096 | -0.03 | 0.97 |
| Left side: medium parasitism | 0 | 0.02 | 3096 | -0.16 | 0.87 |
| Left side: high parasitism | 0 | 0.03 | 3096 | -0.03 | 0.98 |

*significative values, p<0.001.
