# Supplementary material for: Can multitrophic interactions shape morphometry, allometry, and fluctuating asymmetry of seed-feeding insects?
Source: PLoS One. 2020 Nov 11;15(11):e0241913. doi: 10.1371/journal.pone.0241913 (PMC7657534; doi:10.1371/journal.pone.0241913)
Supplement: S7 Table — Results are displayed in comparison to the right side of these structures. (DOCX) [file pone.0241913.s007.docx]

S7 Table. Fluctuating asymmetry between left and right sides for wing and tibia length of *Allorhogas vulgaris,* according to categories of seed biomass, fruit infestation and parasitism rate. Results are displayed in comparison to the right side of these structures.

| *A.vulgaris* |  | Estimate | SD | | d.f. | T | P |
| --- | --- | --- | --- | --- | --- | --- | --- |
|  | **(Intercept)** | 0.53 | 0.01 | 864.01 | | 77.68 | **<0.001*** |
|  | Left side | 0 | 0.01 | 864.01 | | 0.21 | 0.83 |
|  | Medium seed | 0 | 0.01 | 864.01 | | 0.01 | 0.99 |
|  | Small seed | 0.01 | 0.01 | 864.01 | | 1.81 | 0.07 |
| Tibia | Infestation rate | 0.01 | 0.01 | 864.01 | | 1.42 | 0.16 |
|  | Medium parasitism rate | -0.01 | 0.01 | 864.01 | | -1.27 | 0.21 |
|  | High parasitism rate | 0 | 0.01 | 864.01 | | -0.37 | 0.71 |
|  | Left side: medium seed | 0 | 0.01 | 864.01 | | -0.11 | 0.91 |
|  | Left side: small seeds | 0 | 0.01 | 864.01 | | 0.17 | 0.87 |
|  | Left side: infestation rate | 0 | 0.01 | 864.01 | | -0.18 | 0.86 |
|  | Left side: medium parasitism | -0.01 | 0.01 | 864.01 | | -0.75 | 0.45 |
|  |  |  |  |  | |  |  |
|  | **(Intercept)** | 1.72 | 0.02 | 864 | | 81.16 | **<0.001*** |
|  | Left side | 0 | 0.03 | 864 | | -0.01 | 0.99 |
|  | Medium seed | 0.01 | 0.02 | 864 | | 0.72 | 0.47 |
|  | Small seed | 0.05 | 0.02 | 864 | | 2.79 | **0.01*** |
| Wing | Infestation rate | 0.04 | 0.02 | 864 | | 1.82 | 0.07 |
|  | Medium parasitism rate | -0.02 | 0.02 | 864 | | -1.07 | 0.29 |
|  | High parasitism rate | 0.02 | 0.04 | 864 | | 0.39 | 0.70 |
|  | Left side: medium seed | 0 | 0.03 | 864 | | -0.12 | 0.91 |
|  | Left side: small seeds | 0 | 0.03 | 864 | | -0.11 | 0.92 |
|  | Left side: infestation rate | 0 | 0.03 | 864 | | 0.11 | 0.91 |
|  | Left side: medium parasitism | 0 | 0.02 | 864 | | 0.03 | 0.97 |

*significative values, p<0.05.
