# Supplementary material for: Can multitrophic interactions shape morphometry, allometry, and fluctuating asymmetry of seed-feeding insects?
Source: PLoS One. 2020 Nov 11;15(11):e0241913. doi: 10.1371/journal.pone.0241913 (PMC7657534; doi:10.1371/journal.pone.0241913)
Supplement: S8 Table — Results are displayed in comparison to the right elytra. (DOCX) [file pone.0241913.s008.docx]

S8 Table. Fluctuating asymmetry between left and right sides for elytra length of *Stator maculatopygus* according to categories of seed biomass, fruit infestation and parasitism rate. Results are displayed in comparison to the right elytra.

| *S. maculatopygus* X Elytra | Estimate | SD | d.f. | T | P |
| --- | --- | --- | --- | --- | --- |
| **(Intercept)** | 1.16 | 0.02 | 300 | 71.86 | **<0.001*** |
| Left side | 0 | 0.02 | 300 | 0.22 | 0.83 |
| Medium seed | 0.01 | 0.02 | 300 | 0.5 | 0.62 |
| Small seed | -0.01 | 0.02 | 300 | -0.71 | 0.48 |
| Infestation rate | 0.06 | 0.07 | 300 | 0.82 | 0.41 |
| Medium parasitism rate | -0.04 | 0.02 | 300 | -1.66 | 0.10 |
| High parasitism rate | 0.05 | 0.03 | 300 | 1.67 | 0.10 |
| Left side: medium seed | 0 | 0.02 | 300 | 0.02 | 0.98 |
| Left side: small seed | 0 | 0.03 | 300 | 0.09 | 0.93 |
| Left side: Infestation rate | -0.06 | 0.1 | 300 | -0.64 | 0.52 |
| Left side: medium parasitism | 0.01 | 0.03 | 300 | 0.26 | 0.79 |
| Left side: high parasitism | 0 | 0.04 | 300 | 0.02 | 0.98 |
