# Supplementary material for: Can multitrophic interactions shape morphometry, allometry, and fluctuating asymmetry of seed-feeding insects?
Source: PLoS One. 2020 Nov 11;15(11):e0241913. doi: 10.1371/journal.pone.0241913 (PMC7657534; doi:10.1371/journal.pone.0241913)
Supplement: Attachment — Submitted filename: PlosOne-Answers.docx [file pone.0241913.s009.docx]

**Can multitrophic interactions shape morphometry, allometry, and fluctuating asymmetry of seed-feeding insects?**

General comments by the authors:

*We thank the reviewers for the valuable comments on the manuscript. We have revised our manuscript following editor’s suggestion using a word processing program. Indeed, we agreed with most of the suggestions/comments pointed out and have answered point by point the comments raised by both reviewers. The MS English grammar has been revised, and all details about fruit collection were provided in the Materials and methods section. We hope this time expectations are met. Finally, we have organized the comments adding numbers to potential questions so we could answer each point.*

**“Reviewer #1**: Please find below my comments concerning the manuscript by Oliveira et al., entitled: “Can multitrophic interactions shape morphometry, allometry, and fluctuating asymmetry of seed-feeding insects?” (PONE-D-20-22527). The authors present an interesting study regarding to multiple biotic factors that can shape the growth and development patterns of three species with similar ecological functions. This is a seminal work in this specific topic that highlight important aspects the importance of investigating several drivers factors at the same time. The text is well written, the analyses are appropriate, and I think the findings will be interesting for a broader audience. I think the study is sound and I have only some comments or recommendations.”

**Minor comments**

**1-L 25. What are these categories?**

*These categories are fruit infestation, parasitism rate and seed biomass. I did not write them in the abstract because of the limitation of character that we had (250). We changed the abstract.*

*L 24-25. “Despite this, some variations in allometric slopes suggest that interactions in a multitrophic food web can shape the development of these species.”*

**2-L 30-32. This sentence seems inconsistent and / or words are missing. Please, rewrite.**

*I rewrote to:*

*L 29-31. “These findings show that both allometry* ***and*** *morphological traits measurement are the most indicated in studies focused on interactions and morphometry”*

**3-L 42. “create” could be respond through?**

*I changed the word “create” to “influence” as suggested to Reviewer#2*

*L.41-42 “which in turn can* ***influence*** *changes in their fertility, survival, and dispersion [8–10].”*

**4-L 46. Are there any recent references?**

*We added two more recent references.*

*L45.*

*15.	Shingleton AW. Allometry: the study of biological scaling. Nat Educ Knowl. 2010;3: 2. Available: https://www.nature.com/scitable/knowledge/library/allometry-the-study-of-biological-scaling-13228439/*

*16.	Graham JH, Raz S, Hel-Or H, Nevo E. Fluctuating Asymmetry: Methods, Theory, and Applications. Symmetry (Basel). 2010;2: 466–540. doi:10.3390/sym2020466*

**5-L 67-69. Didn't you say that above? Line 64?**

*I know it seems very similar, but they are not the same thing. In the first phrase¹, I talked about body size, and the other phrase² I talked about body patterns, such as allometry and FA. Although we use the morphological structures to evaluate these patterns (allometry and FA), they are three different methods. In allometry, we can see if there is any impact of biotic or abiotic stressors on the relationship between two body parts (here we tested the body weight (beetles) and size (wasp) concerning their body structures (wing, elytra, tibia) In FA, we can see if there is any impact of biotic or abiotic stressors on the symmetry of organism which has bilateral body pattern (such as arthropods). Also, we can simply assess the impact of an abiotic or biotic factor by changes in body size instead of body patterns.
I changed the Abstract to make it clear.*

*L 61-63. “Nevertheless, most studies have analyzed isolated factors influencing the body size of organisms [22,27–33] and few considered multiple interactions affecting body size at the same time”¹*

*L. 66-70 “Likewise, although there are several studies considering isolated factors influencing morphometric patterns (e.g. allometry and fluctuating asymmetry) [19,22,23,25,36], we are not aware of previous studies that have assessed how multiple interactions may or may not affect allometric patterns and (FA)…”²*

**6-L 92: “and for the wasp: right and left wing, right and left tibia length and total body length of A. vulgaris.**

*L 92-93 OK*

**7-L 92. Are there any references considering these measures for wasps?**

*Yes, there are some references. I put these references in the methodology section. However, now that you mentioned I also provided these references in the introduction.*

*L 93“…and for the wasp: right and left wing, right and left tibia length, and total body length of A. vulgaris[43,44].”*

**Methods**
**8-L 108. I missed at least one sentence describing basic information about the plant such as growth form, average height, etc.**

*L 111-117.“Senegalia tenuifolia is locally known as unha-de-gato, and it is a pioneer species largely distributed in South America [46]. S. tenuifolia is characterized as a scandent shrub, i.e. a shrub that is able to become a liana and reach a greater height (up to 8 m) [47]. The flowering period occurs from November to January, and the ripening occurs from January to August (Faria, L.D.B., personal communication). After this phase, fruits open and seeds begin to fall on the ground [45]*”.

**9-L 113-115. Were these fruits collected from the same individuals? If not, how many different individuals? Were the annual collections carried out in the same individuals? Please, provide these important details.**

*We include this in the article:*

L122-124; *“As S. tenuifolia is a liana, we could not distinguish the number of different plants at each site, so we opted to collect the fruits randomly in each subarea [33].”*

**10-L 113. “seven” or eight?**

*Seven, I corrected all information about the areas. Now, I think it is clearer the number of subareas per year Also, as I did not used 2011, I excluded this year in the table and in the text. Further explanation can be found in question number 50.*

*L 102-106 “Across these three areas, we established seven subareas which were at least 400 m from each other (Ae-1: 21°14′5.71′′S- 44°57′8.66′′W; Ae-2: 21°14′7.87′′S-44 58′0.06′′W; La-1: 21°-18′3.46′′S- 44°58′0.53′′W; Lu-1: 21 31′1.36′′S-44°53′1.78′′W; Lu-2: 21 31′5.13′′S-44°52′6.32′′W; Lu-3: 21 31′5.31′′S-44 52′3.84′′W; and Lu-4: 21°41'9.88''S- 44°96'7.18''W); see [45] for additional details of the study site.”*

| Sampling sites and number of fruits per area in each year | | | | |
| --- | --- | --- | --- | --- |
| Areas |  | 2012 | 2013 | 2014 |
|  |  |  |  |  |
| Ae |  | Ae1, Ae2  100 fruits | Ae1, Ae2  99 fruits | Ae1, Ae2  100 fruits |
| La |  | La1  50 fruits | La1  50 fruits | La1  50 fruits |
| Lu |  | Lu1, Lu2, Lu3, Lu4  200 fruits | Lu1, Lu2, Lu3, Lu4  200 fruits | Lu1, Lu2, Lu3, Lu4  200 fruits |

**11-L 180. Why the length and not the width?**

*We could have used any of these measurements (length or width) since they were highly correlated (which means that the result would be the same regardless of the measurement used). We chose length, because in our previously articles we have already standardized this measurement.*

**12-L 238-247. The legend of Figures 4 and 5 looks similar, both in relation to *S. maculatopygus.***

*Well observed, my mistake, I corrected them. Figure 5 is related to A. vulgaris. Also, I examined each figure in detail, and fixed the errors in their labels.*

*L. 266-271 “****Fig 5****: Negative allometry depicted by the slopes and their confidence intervals (CI) for the* ***tibia*** *and wing allometry (****tibia*** *length and wing length in relation to body* ***size****) among infestation, parasitism rate, and seed biomass categories for Allorhogas vulgaris individuals (CI of 95%).* ***Low (0-0.30%), medium (0.31-0.60%), and high (0.61-1%) infestation and parasitism rate (low and medium); and among small, medium, and large seeds.****”*

**13-L 262. Isn't it Figure 3? Please, see my comment above and check this figures citation on MS.**

*L288 You are right, this is figure 3. In the last minute I included picture 2, and I had to change all numbers through the paper, this one I must forget to change. Thank you. I fixed it.*

**Discussion.**
**14-L 338-400. Please, detail how the great variation in allometric slopes can influence in the abundance of individuals.**

*We did not mean that allometric slopes can influence the abundance of the individuals. We meant that both allometric slopes and the insect’s abundance were influenced by the categories (seed biomass, infestation, and parasitism rate). I added this information after the word “variables” to specify which one we talked about:*

L 363- 367 “*The fruit infestation, parasitism rate, and biomass of the resource did not change the morphological allometric pattern of the three seed-feeding insect species. However, as we found a great variation in allometric slopes, it seems these variables* ***(e.g. fruit infestation, fruit parasitism and seed biomass****) can influence the insect's development, both in its allometry and abundance.”*

**15-L 378-379. Did the authors test this?**

*You are right, we did not test it. Therefore, we removed this part from the article.*

*~~“These results suggest that, for these insects, the parasitism rate is a more stressful factor than the rate of infestation. In fact, we observed a low abundance or absence of these insects at the highest levels of parasitism.”~~*

**16-L 388. Maia et al. (2017).**

*We changed all references that were not number in the article.*

**17-L 411. Space before “By contrast …”**

*L 439 Ok*

***Reviewer #2:* Review of manuscript #PONE-D-20-22527 “Can multitrophic interactions shape morphometry, allometry, and fluctuating asymmetry of seed-feeding insects?” by Oliveira et al.**

“General comments and assessment:
The manuscript (henceforth MS) assesses if multitrophic interactions might shape morphometry, allometry, and fluctuating asymmetry of seed-feeding insects in a plant species from Brazilian Cerrado. The study is innovative and brings a great advance to the understanding of how ecological interactions can influence individual performance. I really liked to read this work and I think this is a great contribution.
I can point out two major issues in the MS: there are many typos and English grammar issues. The MS is well structured, but some parts are hard to follow, or totally not understandable. The MS needs to be proofread by a native. I could provide many small issues over the MS, but it would be good to recheck the spelling and grammar.
The second point it is the lack of details in some parts of the methodology, which I highlight bellow. The main issue that I pointed out, it is that the authors did not mention how many plants they used, or how many fruits per plants they collected, which can bias the analyses, eg. spatial correlation, low variability of samples.

**More specific comments:**
Abstract
In my opinion the abstract needs some improvement and clarity to really reflect what was found in MS. I found some typos and issues in English grammar. Also the structure is not good, since nothing about methodology is mentioned. Further, the conclusions do not seem to follow the results. So I recommend to reword some parts and recheck English typos.”

**18-L6, and 8 – Separate city name from country with comma.**

*L6, and L 8 Ok*

**19-L17 – HAVE TRIED… - present perfect, not continuous.**

*L 15 Ok*

**20-L21-22 – You tested the effects of specific variables on morphological structures. In L22-23, you tested the effects of these specific variables on allometric patterns, fluct. asymmetry, and MORPHOLOGICAL STRUCTURE. Doesn’t morphological structure encompass these two other results (allometry and asymmetry)? What I mean it is that you used only morphology before, but later you used morphology, asymmetry, and allometry. So it would be good to standardize and be clear which parameter is different from each other and which can be encompassed by the other. In your conclusion you used only morphology; so I expect that this term encompasses the other two. If I wrong, please be clear in your statements.**

*You are correct the two other tools (allometry and FA) analyze the effect on the morphometric structures, but they do it differently. In allometry we evaluate the growth ratio of one morphological part in relation to another, in fluctuating asymmetry; we evaluate the difference of one side in relation to the other. However, we also assessed whether the categories influenced the size of the morphological structures individually. To improve understanding we removed this part:*

*~~“We tested how these variables affect the allometric patterns, fluctuating asymmetry, and the morphological structures of these seed-feeding insect species”.~~*

*And add this in bold :*

*L18-25 “Here, we evaluate the effect of fruit infestation, parasitism rate, and seed* ***biomass on size, allometric and asymmetric patterns of morphological structures of insects that exploit the same resource.*** *To test it, we measured 750 individuals associated with the plant Senegalia tenuifolia (Fabaceae), previously collected over three consecutive years.* ***Negative allometry was maintained for all species, suggesting that with increasing body size the body structure did not grow proportionally. Despite this, some variations in allometric slopes suggest that interactions in a multitrophic food web can shape the development of these species.”***

**21-L24 – Methodology is missing. I recommend you to add some words about methodology here. Maybe you can add a few words before the sentence “we tested (L. 22)…” telling time and specie used. Like: “For that, analyzing PLANT SPECIES (FAMILY) over four consecutive years, we tested how these FRUIT-RELATED TRAITS affect**…

*L20-22“To test it, we measured 750 individuals associated to the plant Senegalia tenuifolia (Fabaceae) previously collected over three consecutive year.”*

**22-L24-27 – What is a “negative allometric pattern…”? Do you mean that the relationship between the variables fit in your models showed a negative relationship with allometric parameters? If so reword the sentence. It’s hard to follow what you want to say.**

*I rewrote the sentence:*

*L22-24 “Negative allometry was maintained for all species, suggesting that with increasing body size the body structure did not grow proportionally.”*

**23-L27-28 – Again, what is the relationship between variables and**

*Please see question number 22.*

**24-L29 – FOUND**

*L28.OK*

**25-L30-32 – When you say “besides” you give an idea of addition to “allometry”. However, later you mentioned “the most” a superlative, comparative. This is contradictory. Or both parameter, allometry and morphological, are good to evaluate, or one is better than other.**

*L29-31“These findings show that* ***both*** *allometry and morphological trait measurements are the most indicated in studies focused on interactions and morphometry.”*

**26-L31- “allometry, …”**

*I changed the Abstract :*

*L 29-31“These findings show that both allometry and morphological trait measurements are the most indicated in studies focused on interactions and morphometry.”*

**27-L32-34 – According to your results in abstract, I can’t see that each species respond differently. You wrote that you didn’t find fluctuation asymmetry for categories of species. Only that. So I can’t say only reading the abstract that each species respond differently.**

*Yes, you're right. I did not let it clear that the result for FA were the same for all species in the abstract. This because I had a hard time summarizing the article in 250 words, so some information seems to have been missing*.

*I rewrote:*

*L 31-33 “ Finally, we show that,* ***except for the fluctuating asymmetry****, each species and morphological structure respond differently to interactions, even if the individuals play the same functional role within the food web.”*

**Introduction**
**28-L39-40 – change semicolons to commas. Not common in English**.

*L39-40 Ok*

**29-L43 – change “can create” to “influence”**

*L43 Ok*

**30-L44 – change “destinated” to “allocated”**

*L 43 Ok*

**31-L51 – add “FA” abbreviation here, when you say for the first time “fluctuating asymmetry”. Remove “fluctuating asymmetry” from L55 and L71. Use only the abbreviation instead.**

*L 54 and 69 Ok*

**32-L65 – For me it is a little weird mentioning your own study in a third person “Oliveira et al. …”. I would use: Recently, we observed … (Oliveira et al. 2020).**

*L 64 Ok*

**33-L66 – change “;” to “,”.**

*L 65 Ok*

**34-L69 – If you provided an abbreviation for a term (FA), please use it over the MS (there are many other parts with the term and not the abbreviation), and not the term itself.**

*L 68 Ok*

**35-L73-76 – I think this is not your main aim. If you see your abstract, that one is a good and appropriate aim. Provide a general view of your study and not a specific, as you did. You evaluated the effects of different interactions on the morphology of three different insects, which exploit the same plant resource. So this is bigger than what you have provided as aim. Next, you can provide all this information about the system**.

*We rewrote:*

*“Thus, the present study evaluates the effect of different interactions on the morphology of three different insects, which exploit the same plant resource”.*

**36-L76: remove space between “Hymenoptera” and “:”**

*L 75-76 .Ok*

**37-L84 – change “interesting” to “advantageous”**

*L 83-Ok*

**38-L86 – here you can clearly see that there is no relationship between your current aim and what you hypothesized. What is the relationship between “increase in competition and parasitism rate” and your current aim in the Introduction? The answer is none. So, because of that, you need to reword your aim.**

*Ok. Please see question 35.*

**39-L86-89 – For (I), you didn’t mention a specific relationship (positive, negative, or neutral) as for (II), which was “greater”, and (III), which was “reduction”. Why that? I would expect a reduction in allometric patterns.**

*At first I was talking about the allometric pattern (positive (slope values >1), negative (slope value < 1) or neutral (slope=1)), but now that you mentioned, it makes sense to talk about the effect that I was expecting in the insects allometric scaling instead of their pattern. Please see question number 85 and 86 to further explanation about allometric slopes values.*

*. I changed the hypothesis to:*

*L86-89 “We hypothesize that an increase in fruit infestation, parasitism rate, and a reduction in seed biomass cause: (I) a negative change in allometric slopes; (II) greater deviations in the fluctuating asymmetry of these species; (III) and reduction in the size of their morphological structures.”*

**40-L90 – change “explained” to “explain”**

*L 90 Ok*

**41-L91 – change “bruchine beetles for the Coleopteran [19,22]: pronotum” to “bruchine beetles [19,22], namely pronotum”**

*L 91 Ok*

**42-L92-93 – change “for the wasp right and left wing right and left tibia length and total body length of A. vulgaris” to “for the wasp, right and left wing, right and left tibia length, and total body length of *A. vulgaris*”**

*L 92-93Ok*

**43-L93-94 – You mentioned “allometric relationships and FA”, but in other parts you wrote three variables: allometry, FA, and morphology. Please, be consistent with what you are really testing and standardize you MS. See my comments in Abstract about this. Mention one term which encompasses all three, or the three terms.**

*L 93-94“We evaluated* ***size,*** *allometric relationships and the fluctuating asymmetry of these morphological traits according to different…”*

*I changed the abstract and also this part of introduction. I want readers understand that besides allometric and FA patterns, I also evaluated how the categories could change or not the size of morphological traits.*

**44-L94-95 – does “seed infestation” refer to “competition” (L86)? Again, please standardize your MS and do not change the terms over the MS.**

*I decided to change the word “competition” for the word “seed infestation “because it is more conservative and less speculative, and some of them have not been replaced. Thank you for alerting me.*

*I changed all of them and standardize for fruit infestation.*

**Methodology**
**45-L103-106 – the degree symbol is not correct. Remove ‘0’ before degree numbers with two digits (044).**

*L 103-105 Ok*

**46-L106 – Reference is not correctly formatted. “Tuller et al. (2015)”**

L 106 *Ok*

**47-L108 – Inform some characteristic of the plant species studied before saying how you assessed beetles and fruits. Is it a shrub, tree,…? What was the mean size of each plant individual? We need to know some plant traits that make your MS replicable.**

*We included some information of S. tenuifolia:*

*L 111-117“Senegalia tenuifolia is locally known as unha-de-gato, and It is a pioneer species largely distributed in South America [46]. S. tenuifolia is characterized as a scandent shrub, i.e., a shrub that is able to become a liana and reach a greater height (up to 8 m) [47].The flowering period occurs from November to January, and the ripening occurs from January to August (Faria, L.D.B., personal communication). After this phase, fruits open and seeds begin to fall on the ground [45].”*

And…

*L 122-124“As S. tenuifolia is a liana, we could not distinguish the number of different plants at each site, so we opted to collect the fruits randomly in each subarea [33]”*

**48-L111-112 – It is not clear why you removed these data. Low abundance in the same plant, same fruit, or in general? Not clear. Because if you have low abundance in the same fruit/plant is not a reason for removing the data.**

*“You were right when you said to us to remove the year 2011 from the text, as we did not use this year in analyzes. I am not sure if it was clear in the text, but in this work, we used the insects that were previously collected in those determined years by other laboratory members. The insects were stored in the laboratory's collection. For my work, I used the information that already existed in Excel spreadsheets (seed biomass, abundance, parasitoids), and add the information of insects measurements. In 2011, insects were not well preserved, so when I said abundance I referred to the number of individuals in a good preservation state.*

*I excluded: “~~Moreover, we excluded insects collected in 2011 due to the low abundance of the three main seed-feeding insects”~~*

**49-L113 – remove “Therefore”, you are not concluding something.**

*Ok*

**50-L113-122 – In Table 1, there is Lu-4, but not in the text. If you excluded data from 2011, why adding this information in the Table 1? Since you used 2012-2014, you can only focus on these years, and you do not need to mention Ae1 subarea, since it appeared only in 2011. Also you informed La2 in the text, but not in the Table. You need to rewrite all this part of your methodology, including 2.1 subtopic and Table 1. Remove 2011 data, and reword Ae2 and Ae3 to Ae1 and Ae2. Make further modifications accordingly to these suggestions.**

*Thank you, I did not see these mistakes. I corrected all of them as suggested:*

*1-I excluded 2011 and Ae1, and changed Ae2 to Ae1 and Ae3 to Ae2*

*2-I excluded La-2, we did not use plants collected in this subarea in this work.*

*L 103-105 “Ae-1: 21°14′5.71′′S- 44°57′8.66′′W; Ae-2: 21°14′7.87′′S-44 58′0.06′′W; La-1: 21°-18′3.46′′S- 44°58′0.53′′W; Lu-1: 21 31′1.36′′S-44°53′1.78′′W; Lu-2: 21 31′5.13′′S-44°52′6.32′′W; Lu-3: 21 31′5.31′′S-44 52′3.84′′W; and Lu-4: 21°41'9.88''S- 44°96'7.18''W)”*

*L 134*

| Sampling sites and number of fruits per area in each year | | | | |
| --- | --- | --- | --- | --- |
| Areas |  | 2012 | 2013 | 2014 |
|  |  |  |  |  |
| Ae |  | Ae1, Ae2  100 fruits | Ae1, Ae2  99 fruits | Ae1, Ae2  100 fruits |
| La |  | La1  50 fruits | La1  50 fruits | La1  50 fruits |
| Lu |  | Lu1, Lu2, Lu3, Lu4  200 fruits | Lu1, Lu2, Lu3, Lu4  200 fruits | Lu1, Lu2, Lu3, Lu4  200 fruits |

**51-L113 – How many plants did you use? How many plants per subarea? Were the same plants over months and years? How many fruits per plant?**

*L 122-124“As S. tenuifolia is a liana, we could not distinguish the number of different plants at each site, so we opted to collect the fruits randomly in each subarea [33]”*

*It was collected 25 fruits per subarea.*

**51-L118 – Is it possible to give some information about fruit ripening at the moment of collection?**

*Yes, It is:*

*L 114-117“…The flowering period occurs from November to January, and the ripening occurs from January to August (Faria, L.D.B., personal communication). After this phase, fruits open and seeds begin to fall on the ground [45].”*

**and**

*L 118-120 “The S. tenuifolia fruits were previously collected in 2012, 2013, and 2014 in June, July, and August, during the plant’s ripening season. In this stage, plants can be recognized by their brownish color.”*

**52. Table 1 – Depending on the information provided in the text, you don’t need to provide a table, since it will repeat the same information.**

*In my point of view the table provides a little more detail on the distribution of fruits collected by area and subarea compared to the information in the text. I would like to keep the table or move it to the supplementary material. What do you think?*

**53-L1234 – remove “they were”**

L 139 Ok

**54-L128 – How did you assess seed biomass? Did you dry seeds or not? When did you do that? After how many days, weeks, months? Did you open the fruit to *collect seeds or not? Please provide informations about it****.*

*We added:*

*L 1231-133 “…After this, we opened the fruits and assessed the seed biomass by separating them into paper bags and drying at 40°C for 48 h. Subsequently, they were weighed in precision analytical balance in order to obtain the dry biomass.”*

**55-L129-130 – You have already provided full name of species with authors. Please mention only genus abbreviation and epithet**

*L 144-145 Ok*

**56-L131 – Didn’t understand. Did you use Tuller et al. 2015 to collect these data? I can’t follow this message. It seems that you used biomass and abundance data from Tuller, and not from your study. Maybe you collected the data accoding to Tuller methodology. Please clarify that.**

*Please see question number 48. Also we added this:*

*L 142-146“Building upon data previously collected, we gathered information on seed biomass of S. tenuifolia and on the abundance of the three seed-feeding species (M. terani Kingsolver, 1980, S. maculatopygus (Pic, 1930) and A. vulgaris Zaldívar-Riverón & Martínez 2018), and their parasitoids in each fruit [45,48].”*

t
**57-L131 – Wrong citation format for PlosOne.**

*Ok*

**58-L134 – Fig. 1 - This is not a plant species food web, but a plant-insect food web. S… tenuifolia is part of the food web. Do not need to write gray scale unless you will explain something from it.**

*L 149-151 “The Senegalia tenuifolia and related trophic levels. The resource plant represents the first trophic level, the second trophic level comprises the three main seed-feeding species..”*

**59-L139 – change to … “previous studies [citation, according to PlosOne rules]”.**

*L 154* *Ok*

**60-L146 – change “structures measures” to “structure measurements”**

*L 160 Ok*

**61-L147 – change to “width, and both”**

*L 161 Ok*

**62-L147 – change to Fig. 2AB**

*L 161 Ok*

**63-L149 – add space between “)[“**

*L 163-Ok*

**64-L151 – change to Fig. 2C**

*L 165* Ok

**65-L151 – Format the citation according to Journal rules**.

*L 165 Ok*

**66-L155 - change to Fig. 2D**

*L169 I put 2C, because there is no D in the figure.*

**67-L 159 – remove space between “Hymenoptera” and “:”**

*L 172 Ok*

**68-L169 – change to “tested”, and to “and resource size”**

*L 182 Ok*

**69-L169 – Fruit infestation is competition? Be clear. Earlier you mentioned competition and infestation. Standardize your terms.**

*I changed the term competition to fruit infestation. Please see question number 44.*

**70-L169-170 – You mentioned the supplementary here as more details about the effects of some variables on morphometric patterns. However, these two sup. files are only formulas to calculate fruit infestation and parasitism. Also, these formulas can easily be added in the MS by: FIR=HT/SA or FIR=HT.SA-1, where FIR is the fruit infestation rate by seed-feeding insects, HT is the total abundance of herbivorous, SA is the total number of seed per fruit; and FPR=PT.(HT+PT)-1, where FPR is the fruit parasitism rate by both coleopteran and hymenopteran parasitoid present in the fruit, PT is the number total of parasitoids in the fruit, and HT is the abundance total of seed-feeding insects in the fruit (Merobruchus terani, Stator maculatopygus and Allorhogas vulgaris). If you keep the formulas in sup., please add formulas according to the order you mention the variables in the MS, i.e., first infestation, then parasitism formula.**

*L 182 193 As you suggested I bring the formula to the main text:*

**71-L171-173 – change to “We defined three categories for both fruit infestation and parasitism rate (adapted from FORMATED CITATION): “Low”, from 0 to 0.30; “Medium”, from 0.31 to 0.60, and “High” from 0.61 to 1**.

*L 194-195 Ok*

**71-L175-176 – use “from” and “to” to say range of seed size, and not hyphen.**

*L 196-198 Ok*

**72-L174(169) – you used the term seed size, but you are actually using biomass. So standardize your text and say exactly what you are measuring, which seems to be biomass and not size.**

Ok

*I changed all “seed size” in the article to “seed biomass”.*

**73-L175-176 – Use only three decimals.**

*L 196-197 Ok*

**74-L178 and others – Inform which correlation, Spearman, Pearson, Kendall...**

*L 198-201“Preliminary Spearman correlation analysis indicated that the elytra length and width of M. terani (correlation of 0.88) and S. maculatopyus (correlation of 0.77), as well as the anterior wing length and width of A. vulgaris (correlation of 0.89) were highly correlated.”*

**75-L187 – change “;” to “,”**

*L 208 Ok*

**76-L191 – change to “We evaluated the fluctuating asymmetry patterns of the species”**

*L 213 Ok*

**77-L194 – change to “We used mixed models analysis with restricted likelihood (REML),”**

*L 216-217 Ok*

**78-L196 – add comma after “i.e.”. Standardize to “fixed effects” or “fixed terms”**

*L 218-2019* *Ok*

**79-L198 – This paragraph is other part of mixed analysis. So, join this paragraph with the previous one.**

*L 219 Ok*

**Results**
**80-L221 – change to “lower”**

*L 242 Ok*

**81-L222 – Table 2 with space. Provide full name of plant species, and insects as well. Provide range for all variables or none. Change to “Total abundance” only. We already know that it is per species. Suggest LFI, MFI, HFI; LPR, MPR, HPR.**

Ok

*L 2424-246 Table1 2: Total abundance of the three seed-feeding insects, Merobruchus terani, Stator maculatopygus and Allorhogas vulgaris, associated to the plant Senegalia tenuifolia by category of fruit infestation, parasitism and seed biomass.*

| **CATEGORIES** | ***Merobruchus***  ***terani*** | | | ***Stator***  ***maculatopygus*** | | ***Allorhogas vulgaris*** |
| --- | --- | --- | --- | --- | --- | --- |
| Low fruit infestation (LFI) | | | 323 | | 45 | 34 |
| Medium fruit infestation (MFI) | | | 191 | | 4 | 51 |
| High fruit infestation (HFI) | | | 0 | | 0 | 61 |
| Low parasitism rate (LPR) | | | 432 | | 43 | 114 |
| Medium parasitism rate (MPR) | | | 71 | | 4 | 28 |
| High parasitism rate (HPR) | | | 15 | | 3 | 0 |
| Small seed) (SS) | | 183 | | | 8 | 61 |
| Medium seed(MS) | | 181 | | | 11 | 62 |
| Large seed (LS) | | 154 | | | 31 | 23 |
| **Total abundance** | | 534 | | | 70 | 146 |

**82-L227 – Change to “Figs.”**

*L 249Ok*

**83-L230-232 – Why not comparing the slopes among categories using likelihood ratio test? It’s better than saying there is “some kind of effect”.**

*I said “some kind of effect” because it wasn’t enough to change the pattern completely (e.g. change the slope to 1 or bigger than 1). In question number 85 and 86, I explained better the allometry, and after you read it I think it will be clear for you why we used “some kind of effect”. Also, since we only have one value and CI per category, it is not possible to compare than using likelihood ratio test.*

**84-L248-249 – I can see a positive effect of infestation in elytra and pronotum on both beetles and pronotum and wing of the wasp. Why not for elytra and why not for the other beetle species?**

*As we discussed, different morphological structures even in the same individual may have different plasticity in face of the same biotic or abiotic factors. In other words, one morphological structure can increase, and others can decrease face the same adversity. As we observed these differences among species we concluded that even though these species have the same functional role in the food web, their morphological structures responded differently to interactions. To corroborate it, we have observed, from two independent experiments still to be submitted for specialized literature, that tibia is more conservative to change than others parts such as antenna (for aphids) and wings (for parasitoids) even though external stresses were equal.*

*Please see question number 86 to better understand what the allometry slopes values mean.*

**85-L254-256 – How did you know that infestation affected negatively elytra?**

*It was affected negatively because the slope values were less than 1. In this specific case we observed a small, but still, some negative variation in the slope values as well, once the relationship among elytra and body size reduced from 0.15 in low fruit infestation to 0.12 in medium fruit infestations. In other words, this small reduction means that the elytra growth rate in MFI is even smaller in relation to its body size when compared to the LFI. Please see the answer to the next question to clarify what the value of slopes to allometry means in biological terms.*

**86-L248-256 – In this case here, I’m quite sure that if CI does not overlap with slope=0 you have a significant influence of variable on insect parameter, and if it is above will have a positive and bellow a negative effect. Also these comments are to 257-265 and 266-278**.

*Yes, we agree with you. If the CI does not overlap with slope zero we have a significant influence of variable on insect parameter. However, there are three allometric coefficients: positive allometry (hyperalometry), negative allometry (hypoallometry) and isometry. The value of these three coefficients is obtained through statistical analysis that results in angles (slope), showing the rate of variation between the growth of body parts and body size. Positive allometry occurs when the angle value (slope) is> 1, which means that the growth rate of the morphological structures is higher in relation to the body size, the negative allometry is exactly the opposite, that is, the growth rate of morphological structures is lower in relation to body size and the angle value is <1, and isometry is when the growth rate of body structures and body size occur in the same proportion with angle = 1 (SHINGLETON; MIRTH; BATES, 2008).*

*SHINGLETON, A.W.; MIRTH, C.K.; BATES, P.W.* ***Developmental model of static allometry in holometabolous insects****. Proc. R. Soc. Lond. B v.275, p.1875–1885, 2008.*

*To clarify: I used the term negative allometry because of most of the slopes values were less than 1. The only species in which the slopes overlapped 1 was A. vulgaris. In this case, we can say that its morphological structures growth tends to isometry. Also, it’s important to note that I used the words “positive” or “negative” effect / positively or negatively affected to determine if the categories increased or decreased the value of the slopes (scaling) respectively. To prevent wrong interpretation about this, I add this information (increased or decreased allometric slopes) after I used these expressions.*

**87-L287 – Add space after “(DA).”**

*L 314 Ok*

**88-L288 – remove “its”.**

*L 315 Ok*

Discussion
**89-L344 – format citation according to PlosOne rules.**

*L 376 Ok*

**90-L355-358 – This period is hard to follow. Maybe you should divide it or reword.**

*L 384-388“On the other hand, the infestation categories and seed biomass negatively influenced (decreased) the elytra allometry slopes of M. terani. This result is similar to the one obtained in another study, in which it was observed that different morphological structures in the same individual can present different plasticity in face of biotic and abiotic factors.[59].”*

**91-L356 – remove the dot after the species epithet.**

*Ok*

**92-L369 – format citation according to PLosOne rules. Also see L375.**

*L 405Ok*

**93-L376-377 – can’t follow the sentence “of their development of the hosts”**

*L 405-407 “Thus, although the morphological structures grow independently from body size, both the lack of food and the acceleration of their development could have caused these changes in the allometric scale of these seed-feeding insect species.”*

**94-L388 – check citation format**.

*L 416 Ok*

**95-L404 – what “this” refers to? Be clear.**

*L 431-434 “Our results confirm that both body size and morphological structure size are more plastic (higher variability) than changes in their symmetry (FA), since we verified some significant relationships between the measured body structures and the categories (e.g., competition and size of the resource), regardless of the side evaluated.”*

**96-L409 – remove comma after M. terani. Do not separate subject and verb.**

*Ok*

**97-L409-411 – Can’t understand these sentences. Reword it.**

*L437-439“The increase in the M. terani elytra was also observed in another study, in which it was verified that increase of competition generates both bigger elytra and body size for this species [23]”*

**98-L412 – remove comma after species name.**

*L 440 Ok*

**99-L415 – What is “predated” – Do you mean “preyed upon by”?**

*Yes, I do. I changed to:*

*L 442-445“In addition to this, we observed in the laboratory that the* ***seeds preyed upon*** ***by*** *these individuals are almost completely consumed, which would explain this negative relationship between the seed biomass and the body size of A. vulgaris [35].*

**100-L429 – remove “the” before “three”**

*L 447Ok*

**101-L423 – incorrect use of food web. The food web is not of a plant species. The plant species is part of the food web. Reword it.**

*L 459-461“We found that although these species have a similar functional role (consuming seeds) in the food web, their bodies have different ways to respond to these interactions.”*

**102-L433-434 – although their bodies scaling did not change ITS pattern - you are referring to scaling.**

*When I said the pattern did not change, I meant it kept negative allometry for all species. To change this pattern, the slopes should be equal to 1 (isometry) or bigger than one (positive allometry). It didn’t happen, so the pattern stayed the same, what changed was the scaling relationship (allometric slopes).*

**103-L434-435 – “they vary substantially in their allometric slopes at high levels of infestation and competition,”. I can’t know what is they, their,… very hard to follow.**

*L 461-465“Also, although their body scaling did not change its pattern, keeping slope values <1****, the relationship between body size and morphological structures*** *varies substantially at high levels of infestation and fruit infestation (****higher CI values****), demonstrating that this tool can be effectively used in evaluations of this type.”*

**104-L439 - change to “insects’ body”**

*L 467 Ok*

**Acknowledgements
105-If I’m not mistaken there is specific sentence for Brazilian foundation program CAPES.**

*Ok*

**FIGURES
106-In Fig. 1 there is the word FIGURAS at the top of the page. Change to FIGURES.**

*Ok*

**107-Fig 2-7 – Increase the size of number in x- and y-axis. They are very small.**

*Ok, I changed the size of the numbers in x and y-axis, I also carefully examined all figures, some of them were with wrong labels. I fixed all of them.*

**108-Fig. 6 – You add posthoc test in your figure but you didn’t mention it in the analyzes. Did you use Tukey? Estimated marginal means? Also place the letters close to the SE bars.**

*I add it in the text:*

*L 228-229“Significance levels between groups were tested by post-hoc Tukey preventing overestimated inferences with the ‘lsmeans’ package [53]”*

*Also, I placed the letters closer to the bars. We also changed SE to IC in the bars because we decided it is easier to see differences among categories using IC instead of SE.*

**109-Fig. 6-7 – Please do not use red color in MSs since it is not accessible for readers with reduced color vision. It would be grateful if you could change the color.**

*Ok. We changed it.*

**SUPPLEMENTARY
110-Table S1 – in M1 add space before ~**

*Ok*

**111-In M2 remove space after side in random effect**

*Ok*

**112-L26 - Change “biomass seed” to “seed biomass”**

*Ok*
**113-In legends of Figures and Tables provide full name of species. Abbreviate only if you mention earlier in the own legend.**

*Ok*
