# Supplementary material for: Can multitrophic interactions shape morphometry, allometry, and fluctuating asymmetry of seed-feeding insects?
Source: PLoS One. 2020 Nov 11;15(11):e0241913. doi: 10.1371/journal.pone.0241913 (PMC7657534; doi:10.1371/journal.pone.0241913)
Supplement: Attachment — Submitted filename: Response to Reviwers.docx [file pone.0241913.s010.docx]

**General comments by the authors:**

*We thank the reviewers for the valuable comments on the manuscript. We have revised our manuscript following editor’s suggestion using a word processing program. Indeed, we agreed with most of the suggestions/comments pointed out and have changed point by point according to reviewer #2 corrections.*

**4. Have the authors made all data underlying the findings in their manuscript fully available?

The** [**PLOS Data policy**](http://www.plosone.org/static/policies.action#sharing) **requires authors to make all data underlying the findings described in their manuscript fully available without restriction, with rare exception (please refer to the Data Availability Statement in the manuscript PDF file). The data should be provided as part of the manuscript or its supporting information, or deposited to a public repository. For example, in addition to summary statistics, the data points behind means, medians and variance measures should be available. If there are restrictions on publicly sharing data—e.g. participant privacy or use of data from a third party—those must be specified.**

**Reviewer #1: No**

**Reviewer #2: Yes**

*We include the information in the manuscript:*

*L234-235 All relevant data are within the paper and its Supporting Information files.*

**1-L112-113 – Do not use abbreviation for species name when starting a period. So provide full genus name.**

*L 113 Ok*

**2-L113, 209 and elsewhere – after “i.e.” add a comma**

*L113, 208 Ok*

**3-L220 – Add space between period and “The”.**

*L 219 Ok*

**4-L 255 – I think there is a typo when informing the supplementary. After S2 there is a hyphen and comma, and then S4. Check it out.**

*L 255 Ok, I removed the comma.*

**5-L274-280 – Very long sentence. Please divide it in two at least.**

*L 274-279 Ok*

**6-L281 – Remove space after scale.**

*L 281 Ok*

**7-L289 – I believe after MP is a semicolon and not “:”**

*L 289 Ok*

**8-L296 – Change to “…seed). No variations…”**

*L 296 Ok*

**9-L301 – Remove “(medium seed)”, you have already mentioned it in 295. Then use the abbreviation.**

*L 301 OK*

**10-L395 – I think “growths” is not correct. Do you mean: structure growth?**

*L 394 Ok*

**11-L406 – Change to [68,69]. Thus,… The dot is in a wrong place.**

*L 405 Ok*

**12-L415 – I did not understand this sentence. “to changes in the size of their resource,”. First I think it is “change”. And I don’t what is a change in size of a resource. Would it be amount of resource. Please check it out.**

*L 414 “Besides, the values of the elytra and pronotum allometry of S. maculatopygus were close to zero, indicating that the allometry of S. maculatopygus is not a very sensitive parameter to changes in the amount of the resource”*

**13-L 419 – Use abbreviations constantly over the MS. Do not need to write the words again.**

*L 418 Ok*

**14- L426-429 – This sentence is not well written and clear. I suggest “Some studies have suggested that body size is more sensitive to environmental disturbances than FA, since body size of individuals can present a greater plasticity than FA [75–77].”. Please also check what you mentioned before has the same meaning now.**

*L 425-427 Ok*

**15-L453 – change to “are limiting factors”**

*L 451 Ok*

**16-L 466-470. I found very repetitive theses sentences, and also you suggest/recommend studies in all of them. I recommend you to remove the last two sentences or reword them: “We recommend the use of insects’ body size and morphological structures in studies of food web. However, to clarify and better understand these approaches, more studies are necessary.”**

*L 470-472 I removed the last two sentences.*
